# Pharmacodynamic characteristics of lixisenatide once daily versus liraglutide once daily in patients with type 2 diabetes insufficiently controlled on metformin

**DOI:** 10.1111/dom.12076

**Published:** 2013-02-25

**Authors:** C Kapitza, T Forst, H-V Coester, F Poitiers, P Ruus, A Hincelin-Méry

**Affiliations:** 1 Profil Institut für Stoffwechselforschung GmbHNeuss, Germany; 2 Institute for Clinical Research and DevelopmentMainz, Germany; 3 Sanofi R&DChilly-Mazarin, France; 4 Sanofi R&DFrankfurt, Germany

**Keywords:** GLP-1, metformin, pharmacodynamics, receptor, type 2 diabetes

## Abstract

**Aim:**

Assess the pharmacodynamics of lixisenatide once daily (QD) versus liraglutide QD in type 2 diabetes insufficiently controlled on metformin.

**Methods:**

In this 28-day, randomized, open-label, parallel-group, multicentre study (NCT01175473), patients (mean HbA1c 7.3%) received subcutaneous lixisenatide QD (10 µg weeks 1–2, then 20 µg; n = 77) or liraglutide QD (0.6 mg week 1, 1.2 mg week 2, then 1.8 mg; n = 71) 30 min before breakfast. Primary endpoint was change in postprandial plasma glucose (PPG) exposure from baseline to day 28 during a breakfast test meal.

**Results:**

Lixisenatide reduced PPG significantly more than liraglutide [mean change in AUC_0__:30–4:30h_: −12.6 vs. −4.0 h·mmol/L, respectively; p < 0.0001 (0:30 h = start of meal)]. Change in maximum PPG excursion was −3.9 mmol/l vs. −1.4 mmol/l, respectively (p < 0.0001). More lixisenatide-treated patients achieved 2-h PPG <7.8 mmol/l (69% vs. 29%). Changes in fasting plasma glucose were greater with liraglutide (−0.3 vs. −1.3 mmol/l, p < 0.0001). Lixisenatide provided greater decreases in postprandial glucagon (p < 0.05), insulin (p < 0.0001) and C-peptide (p < 0.0001). Mean HbA1c decreased in both treatment groups (from 7.2% to 6.9% with lixisenatide vs. 7.4% to 6.9% with liraglutide) as did body weight (−1.6 kg vs. −2.4 kg, respectively). Overall incidence of adverse events was lower with lixisenatide (55%) versus liraglutide (65%), with no serious events or hypoglycaemia reported.

**Conclusions:**

Once daily prebreakfast lixisenatide provided a significantly greater reduction in PPG (AUC) during a morning test meal versus prebreakfast liraglutide. Lixisenatide provided significant decreases in postprandial insulin, C-peptide (vs. an increase with liraglutide) and glucagon, and better gastrointestinal tolerability than liraglutide.

## Introduction

Loss of postprandial glycaemic control appears to be the first step in the evolution of deteriorating glucose homeostasis in type 2 diabetes, followed by deterioration of glycaemic control during the prebreakfast and postbreakfast periods, in particular 1. The absolute contribution of postprandial glucose to excess hyperglycaemia in type 2 diabetes appears to be fairly constant across differing levels of glycaemic control; however, in relative terms, its contribution becomes increasingly relevant as glycated haemoglobin (HbA1c) decreases 2,3. Thus, targeting fasting plasma glucose (FPG) alone may be insufficient and additional attention to postprandial hyperglycaemia could increase the chances of achieving, rather than simply approaching, recommended HbA1c targets 2–5. Further evidence also suggests that targeting postprandial glucose (PPG) has the potential to have an independent beneficial impact on the risk of diabetes-related complications 5. Consequently, current treatment guidelines provide targets for postprandial glucose, with the American Association of Clinical Endocrinologists/American College of Endocrinology (AACE/ACE) and the International Diabetes Federation (IDF) recommending that 2-h PPG should not exceed 7.8 mmol/l (140 mg/dl), and the American Diabetes Association (ADA) recommending a more modest target of 10.0 mmol/l (180 mg/dl) 5–7.

Glucagon-like peptide-1 (GLP-1) receptor agonists provide significant improvements in HbA1c, and this class of drugs may help to decrease the risk of hypoglycaemia and promote weight loss 8. However, they have widely differing pharmacokinetic and pharmacodynamic profiles 9–13, and the available evidence suggests that individual GLP-1 receptor agonists differ in their ability to target PPG throughout the day when administered using typical dosing regimens 14–16. Shorter-acting GLP-1 receptor agonists (such as lixisenatide) appear to have a marked effect on PPG levels, which is likely due to substantial slowing of gastric emptying, whereas longer-acting GLP-1 receptor agonists (such as liraglutide) appear to affect mainly fasting glucose and do not have any notable effect on gastric emptying in the long term 14,17.

Liraglutide is currently the only once-daily GLP-1 receptor agonist approved for the treatment of type 2 diabetes. In the phase III programme [Liraglutide Effect and Action in Diabetes (LEAD)] trials, liraglutide (1.2 or 1.8 mg doses) provided absolute decreases of −0.6% to −1.5% in HbA1c and −0.7 to −2.4 mmol/l in FPG when used as monotherapy or in combination with metformin, sulphonylureas and/or thiazolidinediones 16,18–22. Lixisenatide is a new selective once-daily GLP-1 receptor agonist that was approved by the European Medicines Agency in 2013 for the treatment of type 2 diabetes for the treatment of type 2 diabetes 9,13,23–28. Phase II/III data show that lixisenatide 20 µg once daily significantly lowers HbA1c and also has a consistent pronounced impact on postprandial hyperglycaemia, with reductions in 2-h glucose excursions of approximately 5 mmol/l relative to placebo during a standardized liquid meal test 24–28. Here, we present the results of a 28-day, randomized, open-label, two-arm, parallel group, multicentre, pharmacodynamic study (http://ClinicalTrials.gov number: NCT01175473) comparing the effects of lixisenatide versus liraglutide during a standardized solid breakfast test on PPG and other metabolic parameters in patients with type 2 diabetes insufficiently controlled on metformin.

## Research Design and Methods

Male and female individuals aged 37–74 years with type 2 diabetes currently receiving a stable dose of metformin (≥1.5 g/day) and with HbA1c between 6.5% and 9.0%, inclusive, were included in this study. Key exclusion criteria were body mass index (BMI) ≤20 or ≥37 kg/m^2^, serious co-morbidities or abnormalities in laboratory tests [including alanine aminotransferase (ALT) more than three times the upper limit of normal (ULN), calcitonin ≥20 pg/ml, amylase and lipase more than three times ULN], clinically relevant history of gastrointestinal disease, use of other oral or injectable glucose-lowering agents other than metformin within 3 months prior to screening and previous treatment with lixisenatide or liraglutide. Owing to the use of background metformin, patients with renal impairment (creatinine clearance <60 ml/min) were also excluded.

This was a randomized, open-label, two-arm, parallel-group study conducted in seven centres in Germany and consisting of a 2-week screening period, followed by a 28-day treatment period. The study was approved by the ethics committees and was conducted in accordance with the Declaration of Helsinki and Good Clinical Practice guidelines. All patients gave written informed consent to participate in the study.

Participants were randomized in a 1 : 1 ratio to receive once-daily treatment with either lixisenatide (n = 77) or liraglutide (n = 71) stratified by study site. Doses of both study drugs were increased to the maintenance dose over 2 weeks and administered subcutaneously 30 min before breakfast. Lixisenatide doses were 10 µg once daily for the first 2 weeks and 20 µg once daily for the last 2 weeks. Liraglutide doses were 0.6 mg once daily for the first week, 1.2 mg once daily for the second week and 1.8 mg once daily for the last 2 weeks. Participants continued treatment with their established dose of metformin.

At baseline (day −1) and day 28, all participants received a standardized breakfast solid test meal (60.6% carbohydrates, 12.4% protein, 26.9% fat, 451 kcal in total, consumed within 15 min; corresponding to a typical European breakfast) 30 min after study drug administration. On the night preceding the breakfast test meal, participants also received a standardized medium glycaemic index dinner (55% carbohydrates, 14% protein, 31% fat, 674 kcal in total) in order to reduce PPG variability the following morning.

### Endpoints and Assessments

Blood sampling for measurement of pharmacodynamic parameters was performed at prespecified timepoints (prior to the standardized breakfast and over a 24-h period following breakfast) on day −1/day 1 and on day 28/29 (after the last dose of study medication). The primary efficacy endpoint was the change from baseline to day 28 in the area under the plasma glucose concentration–time curve in the 4-h period after the start of the standardized breakfast test meal (AUC_0:30–4:30h_; 0:30 h = start of meal). The AUC was calculated using the linear trapezoidal rule and corrected relative to the premeal glucose concentration. Secondary efficacy measures included changes (baseline to day 28) in maximum PPG excursion in the 4-h period after the start of the standardized breakfast test meal, premeal-corrected AUC_0:30–4:30h_ for serum insulin, serum C-peptide and plasma glucagon levels (6-point profile, including premeal value), 24-h plasma glucose (15-point profile) and mean HbA1c.

Safety and tolerability were assessed based on systematic adverse event and serious adverse event reporting and other specific safety information, including symptomatic hypoglycaemia [clinical symptoms with blood glucose <3.3 mmol/l (60 mg/dl) or prompt recovery after oral carbohydrate administration if no plasma glucose measurement was available]. The safety evaluation for vital signs, ECG and clinical laboratory parameters was based on the review of individual values using potentially clinically significant abnormalities criteria and descriptive statistics. Heart rate and systolic/diastolic blood pressure (SBP/DBP) were measured after 10 min rest in the supine resting position on day −2 (baseline), before injection on days 3, 13 and 29, and at the end of study visit (day 35 ± 2). Heart rate was also obtained from ECG measurements under the same conditions on the same days.

### Statistical Analyses

The primary pharmacodynamic analysis was performed on the modified intent-to-treat (mITT) population, which included all patients who received at least one dose of open-label study drug, and had both a baseline assessment and at least one postbaseline of any primary or secondary pharmacodynamic variable. A linear fixed-effects model was used to assess the difference between the lixisenatide and liraglutide groups for change from baseline in corrected glucose AUC_0:30–4:30h_ determined on day 28, with fixed terms for treatment and study site and with the baseline measurement as covariate. The least square (LS) mean estimate [with corresponding two-sided 95% confidence intervals (CI)] of comparison between lixisenatide and liraglutide was obtained using linear contrasts within the model framework. Secondary pharmacodynamic parameters were analysed using a similar model to the primary analysis.

A sample size of 120 participants (60 per group) was calculated as sufficient to detect a difference of 8.3 h·mmol/L (150 h·mg/dL) in the absolute change from baseline in glucose AUC_0:30–4:30h_ between lixisenatide and liraglutide with a power of 90%. This assumed a common standard deviation (s.d.) of 13.9 h·mmol/L (250 h·mg/dL) at a 5% significance level.

All safety analyses were based on the on-treatment phase defined as the time from the first injection up to 3 days after the last injection. The safety population comprised all randomized patients exposed to at least one dose of open-label study drug. Results are presented as mean ± standard error of the mean (s.e.m.) or LS mean ± standard error (s.e.), unless otherwise specified.

## Results

Demographic and baseline characteristics were comparable between the two study groups (Table 1). The majority of patients (97% in both the lixisenatide and liraglutide groups) completed the 28-day treatment period (Figure S1, Supporting Information). Four patients (two in each group) discontinued prematurely, all due to adverse events. In addition, one patient in the liraglutide group was excluded from the pharmacodynamic analysis due to a protocol deviation (premeal blood sampling was delayed until after the start of the meal). The compliance rate in both treatment groups was greater than 99%.

**Table 1:** Demographics and baseline characteristics (safety population)

Variable	Lixisenatide (n = 77)	Liraglutide (n = 71)
Gender (male/female), %	64/36	70/30
Race (Caucasian/Black), %	99/1	100/0
Age, years (mean ± s.d.)	60.5 ± 7.5	59.7 ± 8.5
Duration of diabetes, years [median (range)]	6.7 (1.1, 30.8)	6.7 (1.1, 25.6)
Weight, kg (mean ± s.d.)	91.2 ± 15.3	92.9 ± 16.6
BMI, kg/m^2^ (mean ± s.d.)	31.2 ± 3.9	31.3 ± 4.1
HbA1c, % (mean ± s.d.)	7.20 ± 0.63	7.41 ± 0.81
Duration of metformin treatment, years [median (range)]	5.0 (0.3, 16.6)	4.7 (0.3, 16.8)

BMI, body mass index;HbA1c, glycated haemoglobin; s.d., standard deviation.

### Pharmacodynamics

**Figure 1:**
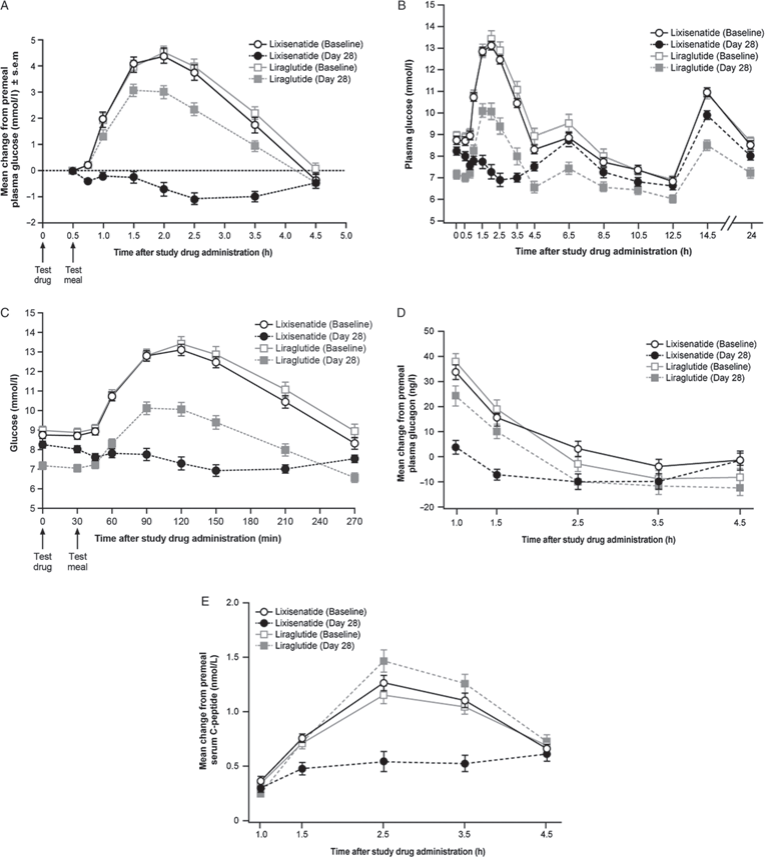
Postprandial plasma glucose pharmacodynamics. (A) Mean ± s.e.m. postprandial plasma glucose change from premeal values at baseline and day 28; (B) Mean ± s.e.m. of raw data for 24-h postprandial plasma glucose profiles at baseline and day 28; (C) Mean ± s.e.m. of raw data for postprandial plasma glucose profiles at baseline and day 28, for the first 270 min after study drug administration; (D) Mean ± s.e.m. plasma postprandial glucagon change from premeal concentration at baseline and day 28; (E) Mean ± s.e.m. postprandial serum C-peptide change from premeal concentration at baseline and day 28; PPG, postprandial plasma glucose; s.e.m., standard error of the mean.

From baseline to day 28, lixisenatide provided a significantly greater reduction in PPG (glucose AUC_0:30–4:30h_ corrected for premeal value) compared with liraglutide (−12.6 vs. −4.0 h·mmol/L, respectively; p < 0.0001) (Figure 1, Table 2). Lixisenatide also provided significantly greater reductions in maximum PPG excursion compared with liraglutide (−3.9 vs. −1.4 mmol/l, respectively; p < 0.0001) (Table 2). From the time–concentration curve for mean PPG at baseline (prior to the start of treatment), it was apparent that the maximum PPG occurred at the timepoint 1.5 h after the start of the meal in both groups (equivalent 2 h after drug administration once treatment commenced).

**Table 2:** Changes in pharmacodynamic parameters from baseline to day 28 or (for fasting plasma glucose) day 29 (modified intent-to-treat population)

Parameter		Lixisenatide (n = 75)	Liraglutide (n = 68)	Estimated treatment difference (95% CI); p value
Glucose AUC_0:30–4:30h_ (h·mmol/l) (primary endpoint)	Baseline	9.40 ± 0.54	10.20 ± 0.68	—
	Mean change from baseline	−12.61 ± 0.55	−4.04 ± 0.57	−8.57 (−10.01, –7.13); p < 0.0001
Maximum PPG excursion (mmol/l)	Baseline	4.89 ± 0.19	4.89 ± 0.24	—
	Mean change from baseline	−3.91 ± 0.21	−1.38 ± 0.21	−2.53 (−3.06, –1.99); p < 0.0001
FPG (mmol/l)	Baseline	8.54 ± 1.58	8.69 ± 1.87	—
	Mean change from baseline	−0.34 ± 0.15	−1.30 ± 0.15	0.96 (0.58, 1.34); p < 0.0001
C-peptide AUC_0:30–4:30h_ (h·nmol/l)	Baseline	3.53 ± 0.17	3.33 ± 0.20	—
	Mean change from baseline	−1.67 ± 0.22	0.35 ± 0.23	−2.02 (−2.59, −1.45); p < 0.0001
Insulin AUC_0:30–4:30h_ (h·pmol/l)	Baseline	714.3 ± 36.1	669.0 ± 49.9	—
	Mean change from baseline	−460.8 ± 50.7	38.3 ± 52.6	−499.1 (−631.3, –366.9); p < 0.0001
Proinsulin AUC_0:30–4:30h_ (h·pmol/l)	Baseline	40.8 ± 5.2	46.9 ± 4.7	—
	Mean change from baseline	−9.1 ± 4.0	−17.7 ± 4.1	8.6 (−1.7, 18.9); p = NS
Glucagon AUC_0:30–4:30h_ (h·ng/l)	Baseline	27.1 ± 7.0	16.5 ± 9.6	—
	Mean change from baseline	−46.7 ± 7.5	−25.3 ± 7.8	−21.4 (−41.0, −1.9); p < 0.05

Data are corrected relative to premeal values. Errors are s.e.m. for baseline values and s.e. for estimated AUC change values. To convert glucose mmol/l to mg/dl, divide by 0.0555; to convert C-peptide nmol/l to ng/ml, divide by 0.333; to convert insulin or proinsulin pmol/l to µIU/ml, divide by 7.175. AUC, area under the curve; CI, confidence interval; FPG, fasting plasma glucose; NS, not statistically significant (p > 0.05); PPG, postprandial glucose; s.e., standard error; s.e.m., standard error of the mean.

A greater proportion of lixisenatide-treated patients (69%) achieved 2-h PPG levels during the breakfast test meal <7.8 mmol/l at day 28 compared with liraglutide (29%). Mean 2-h PPG was 13.1 and 13.4 mmol/l at baseline, and 7.3 and 10.1 mmol/l at day 28 in the lixisenatide and liraglutide groups, respectively.

The 24-h plasma glucose profiles for lixisenatide and liraglutide treatments on day 28 compared with day −1 exhibited an overall reduction in plasma glucose (except before the evening meal at 12.5 h after study drug administration in the lixisenatide group), with decreases in the peak glucose levels that occur in response to meal ingestion (Figure 1B). Plasma glucose profiles on day −1 for the two treatments were comparable. At day 28, plasma glucose levels were much lower with lixisenatide than with liraglutide during the postbreakfast period (i.e., from ∼45 min to ∼4 h after drug administration), whereas from 4.5 h onwards (and before breakfast), plasma glucose levels were lower for liraglutide than for lixisenatide at all timepoints. Both lixisenatide and liraglutide decreased FPG measured 24 h after the last study drug administration, although the effect was significantly greater with liraglutide (−0.3 vs. −1.3 mmol/l; p < 0.0001).

Lixisenatide provided a significantly greater decrease in postprandial glucagon levels from baseline to day 28 (p < 0.05 vs. liraglutide) (Table 2). Postprandial insulin and C-peptide levels were also significantly reduced with lixisenatide versus liraglutide (p < 0.0001 for both parameters), while decreases in pro-insulin were comparable between groups (Table 2). Mean HbA1c decreased in both treatment groups [from 7.2% to 6.9% (−0.32%) with lixisenatide vs. 7.4% to 6.9% (−0.51%) with liraglutide; p < 0.01 for the difference between groups], as did body weight (−1.6 vs. −2.4 kg, respectively; p < 0.01).

### Safety and Tolerability

The overall incidence of adverse events was 58% for lixisenatide vs. 73% for liraglutide. After excluding the preferred term ‘decreased appetite’ (which occurred in 18% of patients on lixisenatide vs. 37% on liraglutide), the incidence of adverse events was still lower with lixisenatide (55%) compared with liraglutide (65%). The difference was mainly due to a lower incidence of gastrointestinal (36% vs. 46%) and nervous system disorders (16% vs. 24%; primarily headache and dizziness) with lixisenatide (Table 3). The most notable difference in individual gastrointestinal disorders (preferred term) was seen for diarrhoea (3% lixisenatide vs. 15% liraglutide). Four patients discontinued due to adverse events – two (2.6%) in the lixisenatide group (one patient with generalized rash/injection site rash and one patient with symptoms related to a suspected allergic reaction, both categorized as moderate) and two (2.8%) in the liraglutide group (one case of severe diarrhoea and other gastrointestinal events in a patient subsequently diagnosed with Crohn's disease and one case of moderate nausea). There were no serious adverse events and no cases of hypoglycaemia reported during the study.

**Table 3:** Safety and tolerability (safety population)

	Lixisenatide (n = 77)	Liraglutide (n = 71)
*Adverse event (AE), n (%)*		
Any AE	45 (58.4)	52 (73.2)
Any AE (excluding decreased appetite)	42 (54.5)	46 (64.8)
Serious AE	0	0
AE leading to death	0	0
AE leading to discontinuation	2 (2.6)	2 (2.8)
Any symptomatic hypoglycaemia*	0	0
Gastrointestinal disorders (any)	28 (36.4)	33 (46.5)
Nausea	17 (22.1)	16 (22.5)
Dyspepsia	6 (7.8)	12 (16.9)
Diarrhoea	2 (2.6)	11 (15.5)
Abdominal distension	5 (6.5)	9 (12.7)
Vomiting	8 (10.4)	5 (7.0)
*Vital sign measurements*		
Δ heart rate, bpm [mean (95% CI)]†	−3.6 (−5.8, –1.3)	5.3 (2.9, 7.7)
Treatment difference, mmHg (95% CI)	−8.9 (−12.2, –5.6)	
Δ ECG heart rate, bpm [mean (95% CI)]†	−3.4 (−5.6, –1.2)	5.9 (3.6, 8.2)
Treatment difference, mmHg (95% CI)	−9.3 (−12.5, –6.1)	
Δ SBP, mmHg [mean (95% CI)]†	−2.0 (−4.9, 0.8)	−2.8 (−5.9, 0.2)
Treatment difference, mmHg (95% CI)	0.8 (−3.3, 5.0)	
Δ DBP, mmHg [mean (95% CI)]†	−0.6 (−2.2, 1.1)	1.1 (−0.7, 2.8)
Treatment difference, mmHg (95% CI)	−1.7 (−4.1, 0.7)	

Δ, change from day −2 (baseline) to day 29; AE, adverse event; bpm, beats per minute; CI, confidence interval; DBP, diastolic blood pressure; SBP, systolic blood pressure.

* Event with clinical symptoms with either plasma glucose <3.3 mmol/l or prompt recovery after oral carbohydrate administration if no plasma glucose measurement was available.

† All measurements taken in the supine position (n = 76 for lixisenatide; n = 68 for liraglutide).

Supine heart rate measured 24 h after the last study drug administration (on day 29) had decreased from baseline by a mean of 3.6 beats/min with lixisenatide versus an increase of 5.3 beats/min with liraglutide, a statistically significant mean difference of 8.9 beats/min (Table 3). Mean heart rate changes based on ECG recordings provided similar findings. Mean changes in SBP and DBP were comparable between the two groups (Table 3). At the follow-up visit (day 35 ± 2 days), all values had returned to baseline levels.

## Discussion

In this study, 28 days of treatment with lixisenatide once daily provided significantly better PPG control during a standardized solid breakfast meal test compared with liraglutide (−129% vs. −41% change in glucose AUC_0:30–4:30h_, respectively). This marked PPG-lowering effect of lixisenatide is consistent with observations from previous studies 9,24–28. The PPG-lowering effect of lixisenatide was associated with significantly greater reductions in postprandial insulin, C-peptide and glucagon compared with liraglutide.

Previous evidence indicates that the glucose-lowering actions of GLP-1 receptor agonists relate to a combination of reduced endogenous glucose production (via insulinotropic and glucagonostatic effects) and reduced appearance of ingested glucose in the systemic circulation (via slowing of gastric emptying), with the latter predominating in the postprandial period 14,29–31. In healthy non-diabetic individuals, glucagon secretion is typically suppressed in response to a meal leading to reduced plasma levels, whereas in type 2 diabetes this suppression is typically impaired 32, as demonstrated by the inappropriately increased glucagon observed before the start of study treatment with lixisenatide or liraglutide.

Insulin secretion was decreased with lixisenatide and relatively unchanged with liraglutide, but this needs to be considered in the context of markedly lower PPG levels with lixisenatide, and these observations would be consistent with glucose-dependent insulinotropic effects and previous observations with exenatide 30,33,34. The decreased insulin secretion seen with lixisenatide in this study would also be consistent with slowing of gastric emptying.

As noted above, the PPG-lowering effects observed with some GLP-1 receptor agonists (lixisenatide and exenatide, but not liraglutide) appear to be due primarily to slowing of gastric emptying 14,17,29–31. Interestingly, recent data indicate that the delay in gastric emptying by GLP-1 is reduced during continued exposure, probably due to tachyphylaxis 35. Such observations suggest that shorter acting GLP-1 receptor agonists, such as lixisenatide and exenatide may have greater potential to reduce PPG due to less tachyphylaxis, and it is possible that such a mechanism also contributed to the greater PPG-lowering effect seen with lixisenatide relative to liraglutide in this study.

Hyperglycaemia associated with the morning meal has been shown to be a fundamental defect that is especially marked in patients with HbA1c levels between 7% and 8% 1. Furthermore, this significant component of overall glycaemia appears to be particularly resistant to glucose-lowering drug therapy, in general 1,36. Postbreakfast glucose excursions, thus represent an important target of glucose-lowering therapy that can be addressed specifically with lixisenatide.

Both lixisenatide and liraglutide were well tolerated and, typical of GLP-1 receptor agonists, the most frequent adverse events were gastrointestinal in nature. However, lixisenatide was associated with a lower incidence of adverse events overall (58% vs. 73% for liraglutide). As the preferred term ‘decreased appetite’ (which was more frequently reported with liraglutide) could be construed as a beneficial effect rather than an adverse event, the tolerability data were also analysed after excluding this term. For this modified total, lixisenatide remained associated with a lower incidence of adverse events overall (55% vs. 65% for liraglutide). In particular, there were fewer gastrointestinal events overall with lixisenatide (36% vs. 46% for liraglutide), and the difference was most notable for diarrhoea, which was five times less frequent with lixisenatide (3% vs. 15% for liraglutide). Gastrointestinal events represent one of the key tolerability issues associated with GLP-1 receptor agonist therapy and can be a major cause of treatment discontinuation in clinical trials, despite their transient nature 8. It remains unclear whether a difference of the magnitude reported in this study would translate into any clinically meaningful advantages, such as improved compliance. However, it is notable that, in a study of patient-reported tolerability issues with oral agents, diarrhoea/constipation was one of only two factors (the other being hypoglycaemia) found to be significantly associated with increased likelihood of self-reported medication non-adherence 37.

Interestingly, mean supine heart rate (measured 24 h after dosing) decreased with lixisenatide, but increased with liraglutide. As both agents provided similar changes in blood pressure, this difference is unlikely to be related to a compensatory mechanism. The increase in heart rate with liraglutide is consistent with previous observations with liraglutide and has also been reported with the weekly formulation of exenatide 16,18–22,38. This observation may warrant further investigation.

Both lixisenatide and liraglutide lowered HbA1c (liraglutide −0.51% vs. lixisenatide −0.32%; p < 0.01) and body weight over 28 days, despite the short study duration. The 24-h profiles showed an overall reduction in plasma glucose in both treatment groups. Specific patterns of coverage appeared to reflect the distinct pharmacokinetic profiles of lixisenatide and liraglutide, with lixisenatide providing particularly good coverage of breakfast-associated glycaemia, as clearly showed in the standardized breakfast meal test, and liraglutide providing better fasting control and PPG coverage beyond the morning meal 9,12. Notably, lixisenatide allowed over twice as many patients to achieve the 2-h PPG target of <7.8 mmol/l (as recommended by the AACE/ACE and the IDF) at breakfast compared with liraglutide. A 2-h postchallenge glucose level of 7.8 mmol/l has generally been recognized as the limit of normal glucose tolerance in healthy individuals and is considered both a reasonable and achievable goal in patients with type 2 diabetes 5. In this study, this goal was achieved by over two thirds of patients on lixisenatide and, importantly, this was possible without any cases of hypoglycaemia. However, lixisenatide also appeared to provide some coverage up to the evening meal, which is consistent with previous observations 9. With respect to clinical reality, a limitation of this study is the relatively short observation time of 28 days. Indeed, direct conclusions with regard to long-term metabolic control should not be made. However, at least with liraglutide, one can expect that the treatment was long enough to reach steady-state conditions, and that in consequence, the full PPG-lowering effect could be achieved 39.

The pronounced PPG-lowering effect of lixisenatide may provide several potential clinical benefits in the treatment of type 2 diabetes. Foremost, lixisenatide would appear to provide a particularly appropriate option for improving glycaemic control in patients with marked postprandial hyperglycaemia. In particular, lixisenatide may represent an alternative option to other PPG-targeting therapies, such as rapid-acting insulin. Notably, lixisenatide's improved control of postprandial hyperglycaemia occurred without increased risk of hypoglycaemia, which can be an issue with rapid-acting insulins. Previous observations also show that lixisenatide itself provides significant improvements in FPG in addition to PPG (e.g. as monotherapy in treatment-naïve patients; [27]). Alongside adequate attention to FPG, targeting PPG with lixisenatide has the potential to allow more patients to achieve (rather than just approach) recommended HbA1c goals. Support for this comes from the prospective interventional study by Woerle et al., who showed that when FPG (but not PPG) was at target, only 64% of patients achieved HbA1c ≤7%, whereas when both FPG and PPG were at target, 94% achieved HbA1c ≤7% 4. Furthermore, targeting FPG with basal insulin in patients insufficiently controlled (HbA1c >7%) on oral agents has been shown to increase markedly the relative contribution of PPG to overall hyperglycaemia from 20–24% to 59–69% across the range of achieved HbA1c (including patients with HbA1c remaining ≥8%), highlighting the need to target PPG on top of FPG if further improvements are to be achieved 40. Epidemiological studies suggest that, even in the absence of fasting hyperglycaemia, elevated postprandial glycaemia increases the risk for cardiovascular disease and death in both diabetic and non-diabetic populations and may also be associated with microvascular complications 5. Thus, specifically targeting PPG may have the potential to reduce the risk of diabetes-related complications 5. Although this has not been confirmed in outcome trials, the benefits of targeting PPG have been demonstrated for surrogate markers of cardiovascular disease (e.g. progression of carotid intima media thickness) 41.

In conclusion, 28 days of treatment with once-daily prebreakfast lixisenatide provided a significantly greater reduction in PPG (AUC) during a morning test meal versus prebreakfast liraglutide. The PPG-lowering effect of lixisenatide was accompanied by significant decreases in postprandial insulin, C-peptide and glucagon, and a better gastrointestinal tolerability profile compared with liraglutide, especially with respect to diarrhoea.

## Supporting Information

**Figure S1.** Patient disposition.
