# Supplementary material for: Pharmacodynamic characteristics of lixisenatide once daily versus liraglutide once daily in patients with type 2 diabetes insufficiently controlled on metformin
Source: Diabetes Obes Metab. 2013 Feb 25;15(7):642–9. doi: 10.1111/dom.12076 (PMC3752965; doi:10.1111/dom.12076)
Supplement: Supplementary file 1 [file dom0015-0642-SD1.doc]

**Supplementary online figure:** Patient disposition

**
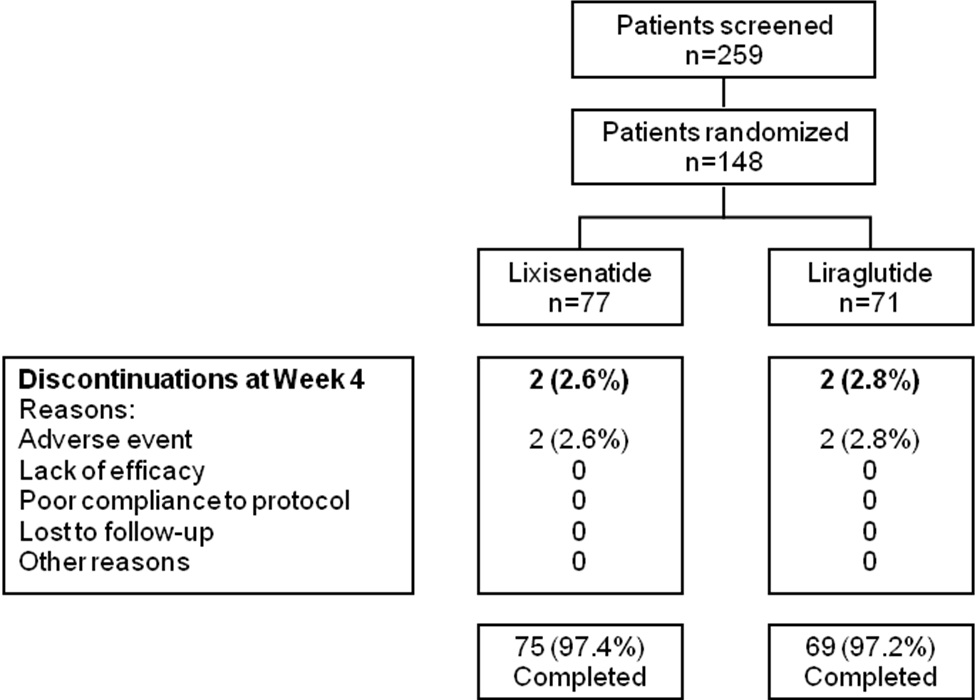
**

One patient randomized to receive liraglutide was excluded from the pharmacodynamic analysis due a deviation in the timing of sampling for the pharmacodynamic parameters.
